# Aridity differentially alters the stability of soil bacterial and fungal networks in coastal and inland areas of Australia

**DOI:** 10.1111/1462-2920.16186

**Published:** 2022-09-16

**Authors:** Qing‐Lin Chen, Qian Xiang, An‐Qi Sun, Hang‐Wei Hu

**Affiliations:** ^1^ Key Laboratory of Urban Environment and Health, Ningbo Urban Environment Observation and Research Station, Institute of Urban Environment Chinese Academy of Sciences Xiamen China; ^2^ Faculty of Veterinary and Agricultural Sciences The University of Melbourne Parkville Victoria Australia; ^3^ Zhejiang Key Laboratory of Urban Environmental Processes and Pollution Control CAS Haixi Industrial Technology Innovation Center in Beilun Ningbo China

## Abstract

Despite the importance of soil bacterial and fungal communities for ecosystem services and human welfare, how their ecological networks respond to climatic aridity have yet been evaluated. Here, we collected soil samples from 47 sites across 2500 km in coastal and inland areas of eastern Australia with contrasting status of aridity. We found that the diversity of both bacteria and fungi significantly differed between inland and coastal soils. Despite the significant differences in soil nutrient availability and stoichiometry between the inland and coastal regions, aridity was the most important predictor of bacterial and fungal community compositions. Aridity has altered the potential microbial migration rates and further impacted the microbial assembly processes by increasing the importance of stochasticity in bacterial and fungal communities. More importantly, ecological network analysis indicated that aridity enhanced the complexity and stability of the bacterial network but reduced that of the fungal network, possibly due to the contrasting impacts of aridity on the community‐level habitat niche breadth and overlaps. Our work paves the way towards a more comprehensive understanding of how climate changes will alter soil microbial communities, which is integral to predicting their long‐term consequences for ecosystem sustainability and resilience to future disturbances.

## INTRODUCTION

Soils harbour highly diverse microbial communities that are crucial drivers of multiple ecosystem functions such as biogeochemical nutrient cycling and plant growth promotion (Fierer, 2017). Soil microorganisms are interconnected via exchanges of information, materials and energy to form complex interactions such as facilitation, mutualisms, competition, and predation (Faust & Raes, 2012; Wan et al., 2022). These interactions are subjected to a diverse array of disturbances with global climate change (Jansson & Hofmockel, 2020). Aridity represents a grand challenge to sustainable crop production and food security and is predicted to increase in intensity and frequency under future climatic scenarios (Greenspan et al., 2020; Song & Haney, 2021). Responses of soil microbial communities and especially their ecological networks to increase in aridity, however, remain largely unexplored, though studies have suggested that changes in network structure will affect ecosystem function and stability (Chen, Wang, et al., 2022; Felipe‐Lucia et al., 2020). Analysing microbial ecological networks will open new avenues to better characterize microbial species interactions and dynamics of ecosystem functions (Guseva et al., 2022; Hernandez et al., 2021).

Despite the critical importance of biological interactions for ecosystem functions, we currently lack a mechanistic understanding of how microbial networks are altered by environmental disturbances. Firstly, previous studies of microbial network patterns have mostly focussed on the soil bacterial communities (Ling et al., 2016; Yuan et al., 2021). For example, a recent field‐based study with multiple long‐term treatments of warming and precipitation alteration found that warming increased the robustness and stability of bacterial networks and strengthened the relationships between network structure with community functional potentials and key ecosystem functions (Yuan et al., 2021). These studies provided valuable information for the relationships between network complexity and stability and shed light on the microbial network dynamics under future climate scenarios. Different groups of the soil microbiomes, however, differ in their responses to disturbances, due to the differences in the plasticity in metabolic abilities and niche breadth (Langer et al., 2019). Therefore, the stability of microbial networks under a given biotic/abiotic stress could depend on the targeted microbial group (de Vries et al., 2018). Secondly, majority of previous studies were conducted under stimulated conditions (de Vries et al., 2018; Yuan et al., 2021), which allowing direct examination of climate extremes on microbial dynamics with less noise variables but overlooking other potential key predictors of microbial communities. Moreover, the differences in parameter settings in these studies make it challenging to provide a mechanistic understanding of inconsistent observations (de Vries et al., 2018; Yuan et al., 2021). Additionally, previous studies were mainly focused on the network complexity such as network size, connectivity, connectance, modularity and number of keystone species (Barberan et al., 2012; Ma et al., 2016), while the network stability has been seldom considered due to lack of proper characterization algorithm. Despite growing interest in microbial network dynamics across space and time, we still lack an adequate understanding of how soil microbial networks of different groups (e.g. bacteria and fungi) shifts under the changing environment. This represents a critical knowledge gap in predicting the responses of microbial ecological patterns to the changing environment and their consequences for ecosystem functions.

**FIGURE 1:**
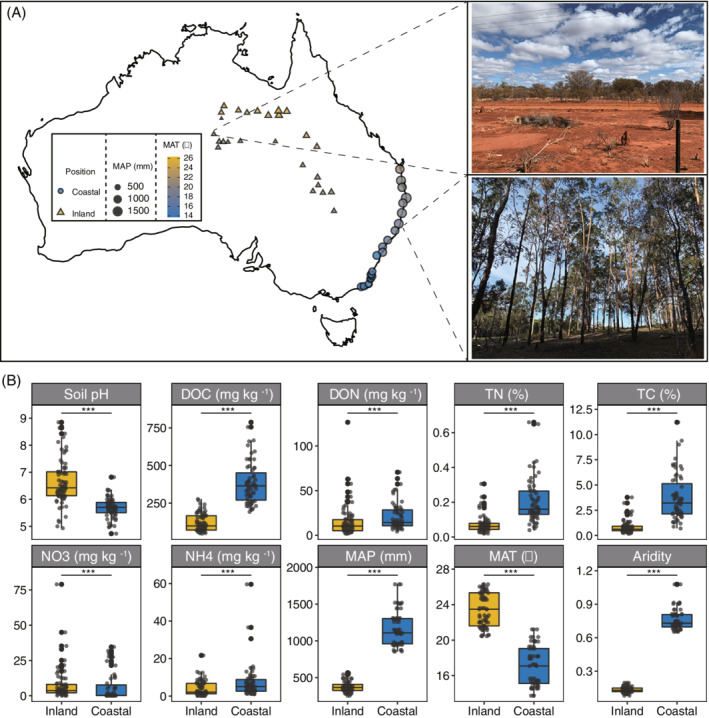
Investigated sites and background information. (A) Sampling locations (*n* = 47), mean annual precipitation (MAP) and mean annual temperature (MAT) of each site, photographs of a representative inland and coastal ecosystem types; (B) comparison of edaphic and climatic attributes between inland and coastal regions. ***p < 0.001. DOC, dissolved organic carbon; DON, dissolved organic nitrogen; MAT, mean annual temperature; MAP, mean annual precipitation; NO_3_, nitrate nitrogen; NH_4_, ammonium nitrogen; TC, total carbon; TN, total nitrogen

In this study, we conducted a large‐scale (~2500 km) field sampling campaign (Figure 1) across 47 locations in humid coastal (aridity index > 0.65) and arid inland (0.03 < aridity index < 0.2) regions of eastern Australia (Trabucco & Zomer, 2018). The aims of the present study were to (i) evaluate whether and how microbial (bacterial and fungal) network stability change from coastal to inland soils and (ii) to compare and uncover the mechanisms underlying the differences in network stability between bacteria and fungi. Given the higher potential migration rates of bacteria than that of fungi, stochastic processes may play a more important role in bacterial community assembly (Chen et al., 2021). Ecological selection under the changing environmental conditions may have less impact on bacteria than on fungi. Therefore, we hypothesize that the bacterial network might be more stable from coastal to inland regions than the fungal network.

## EXPERIMENTAL PROCEDURES

### Field sampling

The soil samples were collected in May 2019 from 47 locations spanning >2500 km across the eastern and northern Australia (133.42° E to 153.60° E, 19.40° S to 37.62° S) (Figure 1). At each site, we established a 10 × 10 m plot in which three subplots were established. Three replicates were collected from each plot by mixing five soil cores (0–10 cm) into one composite sample in each subplot. A total of 141 soil samples were collected and transported to the laboratory on dry ice. Upon arrival, the soil samples were sieved (<2 mm) and divided into two portions. One portion for physicochemical characterization and was air‐dried, and the other portion for molecular analysis was stored at −20°C.

### Climatic data collection

Mean annual precipitation (MAP), mean annual temperature (MAT) and aridity index of each sampling site were obtained from the WorldClim database version 2 (http://www.worldclim.org/) and the Global Aridity Index and Potential Evapotranspiration in Climate Database v2 (https://cgiarcsi.community/) based on the spatial geographical coordinates. MAP and MAT of the sampling area ranged from 264 to 565 mm (20.45–26.29°C) in inland region and 856 to 1769 mm (13.73–21.61°C) coastal regions. Based on the global aridity index classification, the 26 inland locations belong to the climate class of arid (0.03 < aridity index < 0.2) and 21 coastal locations belong to the climate class of humid (aridity index > 0.65) (Trabucco & Zomer, 2018). The detailed information of the climatic attributes of each location was provided in Table S1.

### Soil physicochemical characterization

Soil pH was measured in a 1: 2.5 (M/V) of soil and water suspension with a pH metre (Thermo Scientific Inc., Waltham MA, USA). Soil total carbon (TC) and total nitrogen (TN) were measured using the Dumas combustion method on LECO FP628 analyser (LECO Corp., MI, USA). Soil dissolved organic carbon (DOC) and nitrogen (DON) were extracted with MilliQ water and measured with a TOC analyser (Shimadzu, Kyoto, Japan). Mineral nitrogen including nitrate nitrogen (NO_3_^−^—N) and ammonium nitrogen (NH_4_^+^—N) were extracted with KCl solution (2 M) and measured using the flow analyser (Skalar Analytical B. V. Tinstraat, Breda, Netherland) (Sun, Jiao, Chen, Trivedi, et al., 2021). The detailed results of the soil properties were provided in Table S1.

### DNA extraction and characterization of microbial community structure

Total soil genomic DNA was extracted using the DNeasy PowerSoil DNA Isolation kit (QIAGEN Pty Ltd., Hilden, Germany) *as per* the manufacturer's instructions. The DNA quality was checked using 1.0% agarose gel electrophoresis and the DNA concentration was assessed using a NanoDrop One spectrophotometer (Thermo Fisher Scientific Inc) (Sun, Jiao, Chen, Wu, et al., 2021).

To characterize the bacterial and fungal communities, 16S rRNA gene and ITS region were amplified with the primers 515FmodF/806RmodR (Walters et al., 2016) and ITS1F/ITS2R (White et al., 1990), respectively. Amplicons were purified, pooled at the same concentration, and then sequenced on an Illumina MiSeq PE300 platform (Illumina Inc., CA, USA) at the Majorbio (Shanghai, China). To guarantee the quality of downstream analyses, raw pair‐end reads that containing over three ambiguous nucleotides, reads with a low quality (Q < 20), and short reads (<100 nt) were discarded to generate the high‐quality sequences. The generated high‐quality sequences were analysed and processed using Quantitative Insight into Microbial Ecology (Caporaso et al., 2010). Operational taxonomic units (OTUs) were picked at 3% dissimilarity level using UCLUST clustering algorithm (Edgar, 2010). Representative sequences were assigned to taxonomic lineages against the SILVA database for bacteria (Quast et al., 2012) and UNITE database for fungi (Nilsson et al., 2018).

### Statistical analysis

The statistical analyses of this work were performed in the R (R Core Team, 2016). The Bray–Curtis distance‐based PCoA was conducted to evaluate changes in bacterial and fungal beta‐diversity. Adonis test was performed with the ‘vegan’ package (Oksanen et al., 2017) to further determine the significance in the difference bacterial and fungal beta‐diversity between inland and coastal soils. Random Forest model was employed to identify the important predictors of bacterial and fungal communities compositions with the ‘randomForest’ package (Liaw & Wiener, 2002). The pairwise geographic distance was calculated using the ‘sp’ package (Pebesma & Bivand, 2005) based on the longitude and latitude coordinates. The distance–decay patterns of bacteria and fungi were calculated by the ordinary least‐squares regressions between community similarity (1–dissimilarity of the Bray–Curtis matrices) and geographic distance. We calculated Levins' niche breadth index and Levins' niche overlap for bacterial and fungal taxa with the ‘niche.width’ and ‘niche.overlap ‘functions’, in the ‘spaa’ package (Zhang et al., 2016). We used the neutral community model (NCM) to assess the potential importance of stochastic processes in community assembly (Sloan et al., 2006). We employed the normalized stochasticity ratio (NST) to further quantify the relative importance of determinism and stochasticity (Ning et al., 2019). Network analysis was based on the significant (FDR‐corrected, *p* < 0.01) and strong (*ρ* > 0.6) Spearman rank correlations (de Vries et al., 2018) and visualized by Gephi (v0.9.2). To estimate the stability of the constructed networks, network robustness (Yuan et al., 2021) and proportion of negative links were calculated.

## RESULTS AND DISCUSSION

### Comparison of edaphic, climatic and biotic attributes between inland and coastal regions

**FIGURE 2:**
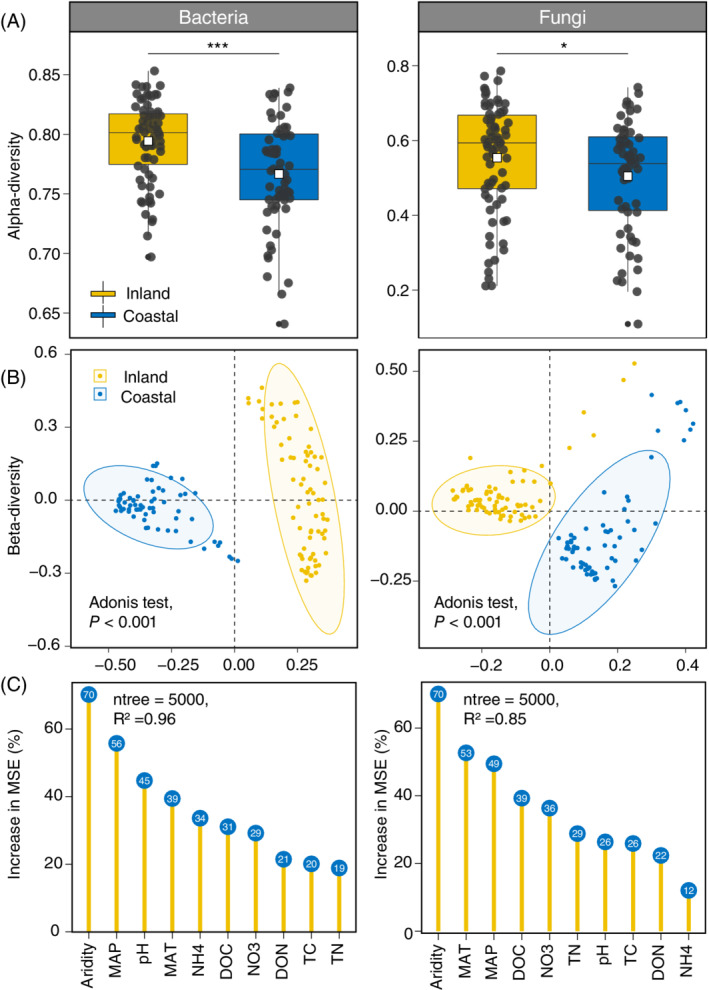
The diversity and predictors of bacterial and fungal communities in arid inland and humid coastal soils. (A) Boxplots of the comparison of bacterial and fungal alpha‐diversity (Pielou's evenness index) between arid inland and humid coastal soils; (B) principal coordinates analysis (PCoA) showing the distinct distribution patterns of bacterial and fungal communities between the arid inland and humid coastal soils; (C), random forest model showing the importance of climatic and edaphic factors for bacterial and fungal communities. *p < 0.05, ***p < 0.001. DOC, dissolved organic carbon; DON, dissolved organic nitrogen; MAT, mean annual temperature; MAP, mean annual precipitation; NO_3_, nitrate nitrogen; NH_4_, ammonium nitrogen; TC, total carbon; TN, total nitrogen

Amplicon sequencing of the 16S ribosomal RNA (16S rRNA) gene and internal transcribed spacer (ITS) region was carried out to examine the compositions of bacteria and fungi in soil samples, respectively. The α‐diversity (Pielou's evenness index) of both bacteria (*p* < 0.001) and fungi (*p* < 0.05) significantly higher in inland soils than in coastal soils (Figure 2A). Similar results were observed for the Shannon index of bacteria, but no significant difference was observed for that of fungi from coastal to inland soils (Figure S1). Principal coordinates analysis (PCoA) showed that the overall community compositions of bacteria and fungi were distinctly separated between inland and coastal regions (Figure 2B), which was corroborated by the nonparametric dissimilarity test of Adonis (*p* < 0.001). We observed significant decay patterns of community similarity with geographic distance for both bacterial (*R* ^2^ = 0.38, *p* < 0.001) and fungal communities (*R* ^2^ = 0.20, *p* < 0.001) and with a steeper slope for bacteria than for fungi (Figure S2). These results suggested that the variation in soil bacterial diversity across space was higher than that of fungi, which was consistent with a previous report that soil heterogeneity significantly influenced bacterial richness but had a minor impact on fungal richness (Seaton et al., 2020).

### Multiple drivers accounting for the bacterial and fungal biogeographic patterns

Soil pH and nutrient availability have been suggested as major drivers of soil bacterial and fungal communities (Delgado‐Baquerizo et al., 2017; Lauber et al., 2009). Despite the significant difference in soil pH and nutrient availability between the inland and coastal soils (Figure 1B), the Random Forest model showed that soil nutrient availability and pH played relatively less important roles compared with climatic factors including aridity, mean annual temperature (MAT) and precipitation (MAP) (Figure 2C). More importantly, we found that aridity was the most important predictor for both bacterial and fungal communities, and the top three major predictors for fungal communities were all climatic attributes. Our previous study also suggested that aridity was the best predictor of soil protistan community (Chen, Hu, et al., 2022). Additionally, a global topsoil microbiome investigation reported similar results that MAP was the major driver of the ratio of bacteria and fungi (Bahram et al., 2018).

### Comparison of microbial assembly processes between inland and coastal regions

**FIGURE 3:**
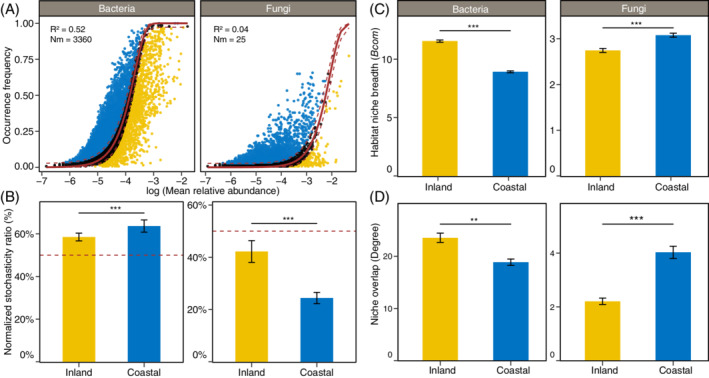
The microbial assembly process, niche breadth and overlap of bacteria and fungi between arid inland and humid coastal soils. (A) Bacterial and fungal communities fitted to the neutral community model showing the OTUs predicted occurrence frequencies versus the relative abundance. (B) Comparison of normalized stochasticity ratio (NST) of bacterial and fungal communities between arid inland and humid coastal soils. (C) Comparison of habitat niche breadth from bacterial and fungal taxa at the community level (*Bcom*) between arid inland and humid coastal soils. (D) Comparison of habitat overlap, that is, the average degree from the networks (Figure S5) between arid inland and humid coastal soils. **p < 0.01, ***p < 0.001

We fitted the soil bacterial and fungal communities data to the NCM and found that the explained variation of NCM for bacteria (*R* ^2^ = 0.52) was higher than that for fungi (*R* ^2^ = 0.04) (Figure 3A). These findings are in agreement with a recent study that compared the role of species sorting and dispersal limitation in agricultural soil microbiomes and found that the balance between species sorting and dispersal limitation mediates soil microbial coexistence patterns (Jiao et al., 2020). The NST was further employed to quantify the role of deterministic and stochastic processes in soil microbial community assembly (Ning et al., 2019) (Figure 3B). The NST value for bacteria was beyond the boundary point (50%), while a NST value < 50% was observed for fungi. These results from the NCM and NST consistently indicated that soil bacterial community assembly was dominated by stochastic process, while deterministic process played a more important role in soil fungal community assembly. A previous study also suggested that soil bacterial communities were governed more strongly by ecological stochasticity relative to ecological determinism than protistan communities (Wu et al., 2018). The ‘size‐plasticity’ hypothesis assumed that smaller organisms (e.g. bacteria) are less environment filtered than larger organisms (e.g. fungi), as smaller organisms are more likely to be plastic in metabolic abilities and have greater environmental tolerance (De Bie et al., 2012). This was supported by the observation of a higher community‐level habitat niche breadth (*Bcom*) of bacteria than that of fungi (Figure 3C). Therefore, the role of deterministic process in microbial community assembly tends to be increase with organism size (Farjalla et al., 2012). Moreover, ecological stochasticity of bacterial communities was significantly higher in more humid coastal soils than in arid inland region (*p* < 0.001), while ecological determinism significantly increased for fungal community assembly (*p* < 0.001) (Figure 3B). As the complement of previous findings that soil pH and precipitation mediated the balance between stochastic and deterministic assembly of bacteria (Tripathi et al., 2018; Yang et al., 2022), our observations further indicated that aridity could also differently balance ecological stochasticity and determinism of bacterial and fungal communities assembly from coastal to inland regions. In addition, our results suggested that aridity contrastingly shifted the bacterial and fungal *Bcom* (Figure 3C) and niche overlaps (Figure 2D, S3), which could be partially explained the distinct responses of bacterial and fungal communities assembly processes to aridity.

### Comparison of microbial networks topological features between inland and coastal regions

**FIGURE 4:**
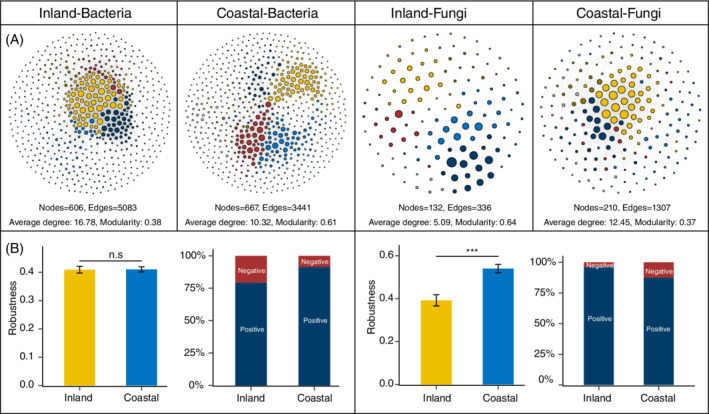
The complexity and stability of microbial networks. (A) Network analysis shows bacterial and fungal co‐occurrence patterns in arid inland and humid coastal soils; (B) the stability of network as determined by robustness and proportion of negative links within each network. n.s *p* > 0.05, *** *p* < 0.001

We further compared the bacterial and fungal networks between inland and coastal regions (Figure 4) on the basis of significant and strong Spearman correlations (de Vries et al., 2018). For bacterial networks, we observed decreasing in node degree and closeness from inland to coastal soils (Figure S4), while a higher modularity indicated a higher clustering bacterial network in more humid coastal soils (Figure 4A). Fungal networks showed lower node degree and closeness in more arid regions (Figure S4), with a higher clustering in more arid inland soils (Figure 4B). All these observations suggested that aridity could increase the complexity of bacterial networks, while decreasing that of fungal networks.

In addition to the network complexity, we further assessed the stability of the bacterial and fungal networks, by randomly removing nodes from the networks to simulate species extinction and calculate network robustness (Yuan et al., 2021). We found that bacterial network robustness showed no significant difference between inland and coastal soils (*p* = 0.421). By comparison, a higher robustness (*p* < 0.001) was observed for fungal networks in humid coastal than in arid inland regions (Figure 4B). In consistence with patterns of fungal networks, our previous work also found the similar results for protistan networks (Chen, Hu, et al., 2022). In addition, we compared the proportion of negative and positive microbial correlations (Figure 3B). It has been suggested that the presence of negative links could increase the stability of networks under environmental fluctuations (Coyte et al., 2015). Cooperation (positive relationship) can destabilize ecological networks by causing dependencies, whereas competition (negative relationship) can be stabilizing because it creates negative feedback between species and thus stabilizes co‐oscillation and trophic cascades (Coyte et al., 2015; Palmer Jacob & Foster Kevin, 2022). We found that negative links decreased from arid inland region (20.7%) to humid coastal region (8.6%) in bacterial networks. Contrastingly, negative links in fungal networks increased from arid inland region (3.9%) to humid coastal region (12.5%) (Figure 4B). As negative links could reflect the degree of niche overlap including the competition for limiting resources as well as environmental spaces. These results were also supported by the results that bacterial community niche overlap tended to increase from the coastal to inland regions, while fungal community overlap showed an opposite pattern (Figure 3D). Collectively, based on the complexity, robustness and proportion of negative links in bacterial and fungal networks, our results suggested that aridity could differentially alter the bacterial and fungal networks, as bacterial networks tend to be more stable in more arid regions, while fungal networks tend to be more stable in more humid region. These findings challenge the previous thought that fungal networks are more stable than bacterial networks under the drought stress (de Vries et al., 2018).

## CONCLUSIONS

Together, our work provides a novel evidence that bacterial and fungal communities traits including community level niche overlap, assembly processes and ecological network stability showed distinct responses to aridity (from coastal to inland regions). These findings might have profound implications for projecting how soil bacterial and fungal networks and ecological consequences respond to the changing environment. Given the crucial roles of bacteria and fungi as well as their ecological networks in maintaining ecosystem functions, preserving microbial networks (‘interactions’) should be taken into consideration in future ecosystem conservation strategies.

## CONFLICT OF INTEREST

The author declares that there is no conflict of interest that could be perceived as prejudicing the impartiality of the research reported.

## Supporting information


**Appendix S1** Supporting Information.

**Table S1** Supporting Information

## Data Availability

The raw sequence has been deposited in the Sequence Read Archive of the National Centre for Biotechnology Information with the accession number PRJNA659980.
