# Supplementary material for: Aridity differentially alters the stability of soil bacterial and fungal networks in coastal and inland areas of Australia
Source: Environ Microbiol. 2022 Sep 16;24(11):5574–82. doi: 10.1111/1462-2920.16186 (PMC9825871; doi:10.1111/1462-2920.16186)
Supplement: Supplementary file 1 — Appendix S1 Supporting Information. [file EMI-24-5574-s002.docx]

# Aridity differently alters the stability of soil bacterial and fungal networks

Qing-Lin Chen ^1,2^, Qian Xiang ^1,3^, An-Qi Sun ^1,3^, Hang-Wei Hu ^2,^*

^1^ Key Laboratory of Urban Environment and Health, Ningbo Urban Environment Observation and Research Station, Institute of Urban Environment, Chinese Academy of Sciences, Xiamen 361021, China.

^2^ Faculty of Veterinary and Agricultural Sciences, The University of Melbourne, Parkville, VIC 3010, Australia.

^3^ Zhejiang Key Laboratory of Urban Environmental Processes and Pollution Control, CAS Haixi Industrial Technology Innovation Center in Beilun, Ningbo 315830, China.

*Corresponding author: hang-wei.hu@unimelb.edu.au (H.-W. Hu).

**Running title**:

Aridity alters soil microbial networks


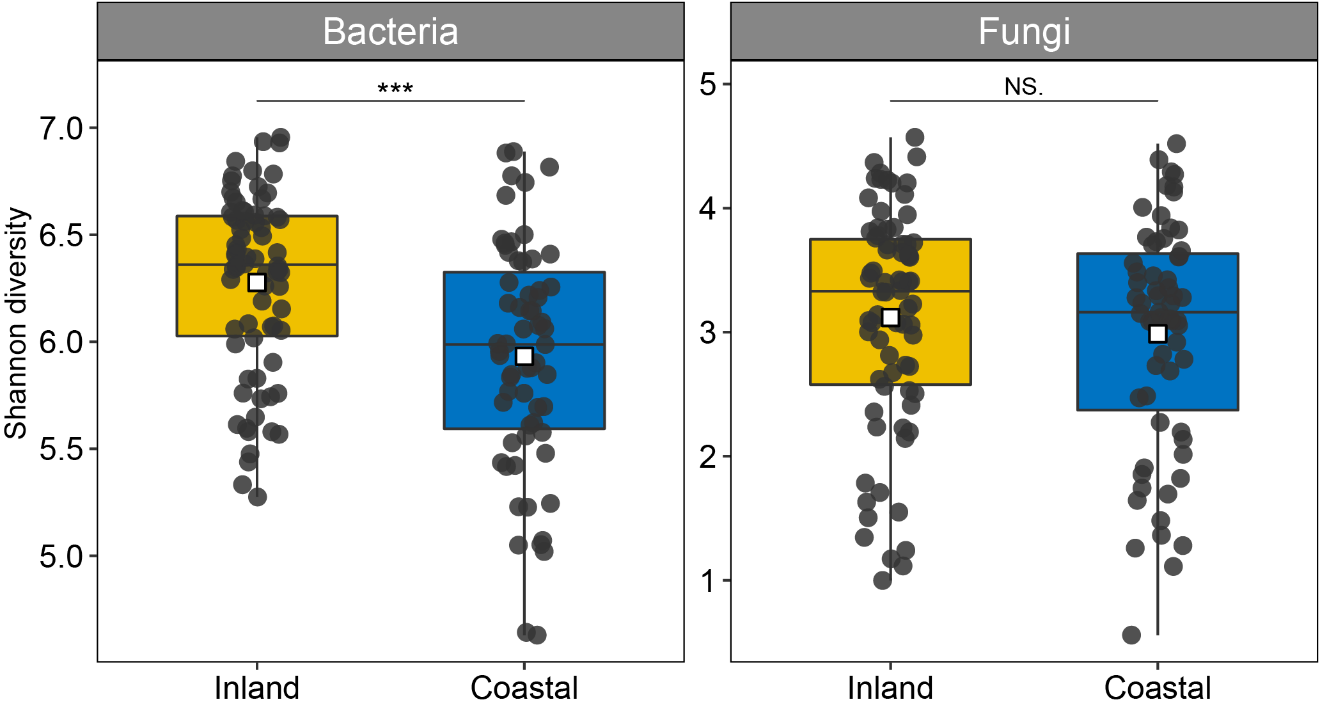


Fig. S1. Comparison of bacterial and fungal Shannon diversity between inland and coastal regions. N.S *P* > 0.5, *** *P* < 0.001.


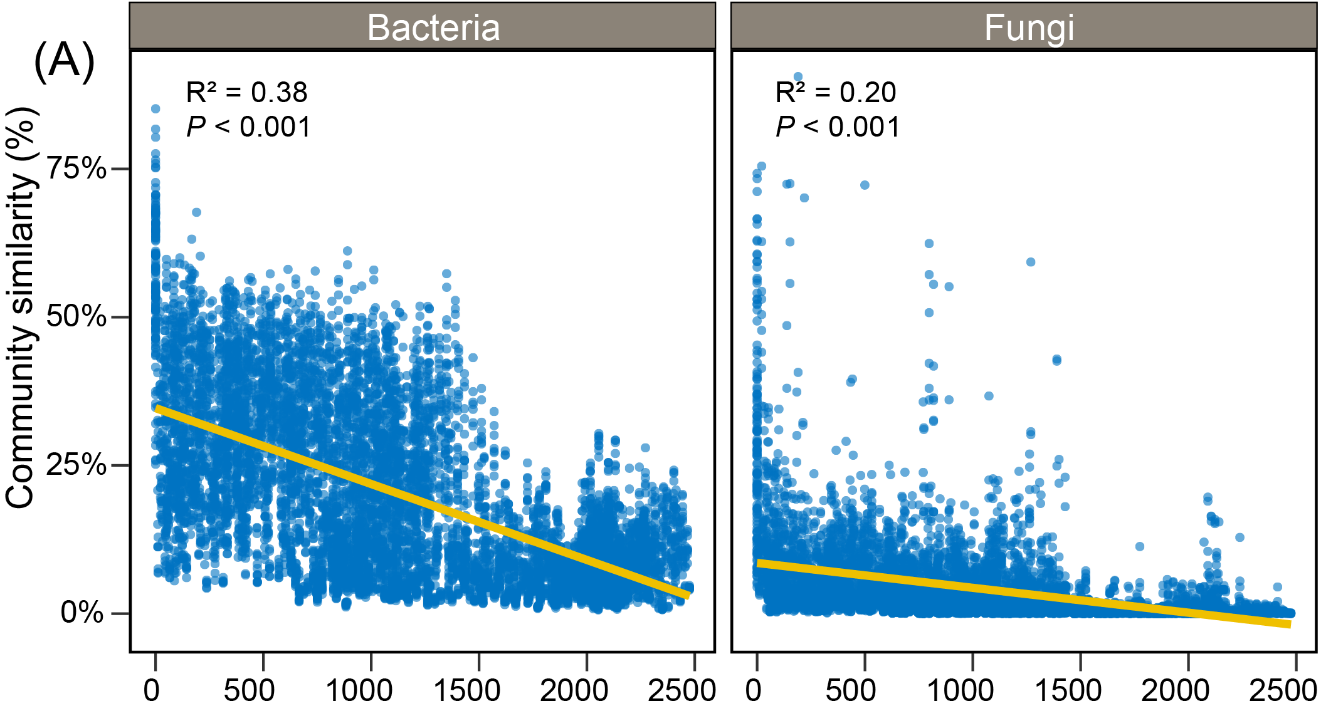


Fig. S2. Distance-decay curves showing the relationships between the Bray–Curtis similarity of bacterial and fungal communities and geographic distances between sampling sites.


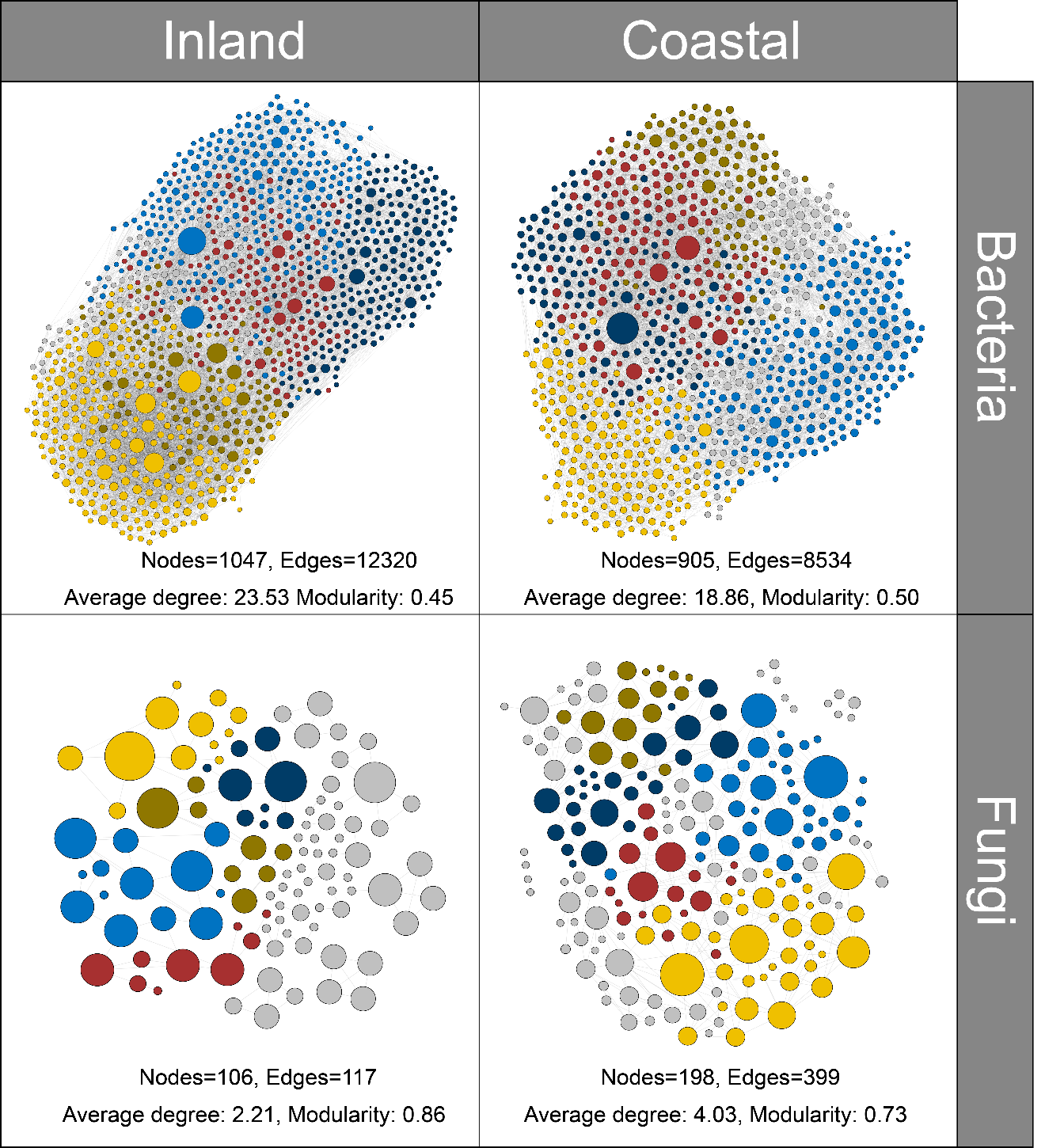


Fig. S3 Network analysis shows the bacterial and fungal niche overlap patterns in inland and coastal regions (A). Nodes represent individual OTUs; edges represent the value of Levins’ niche overlap. Top five major modules are shown in different colours, and smaller modules are shown in grey.


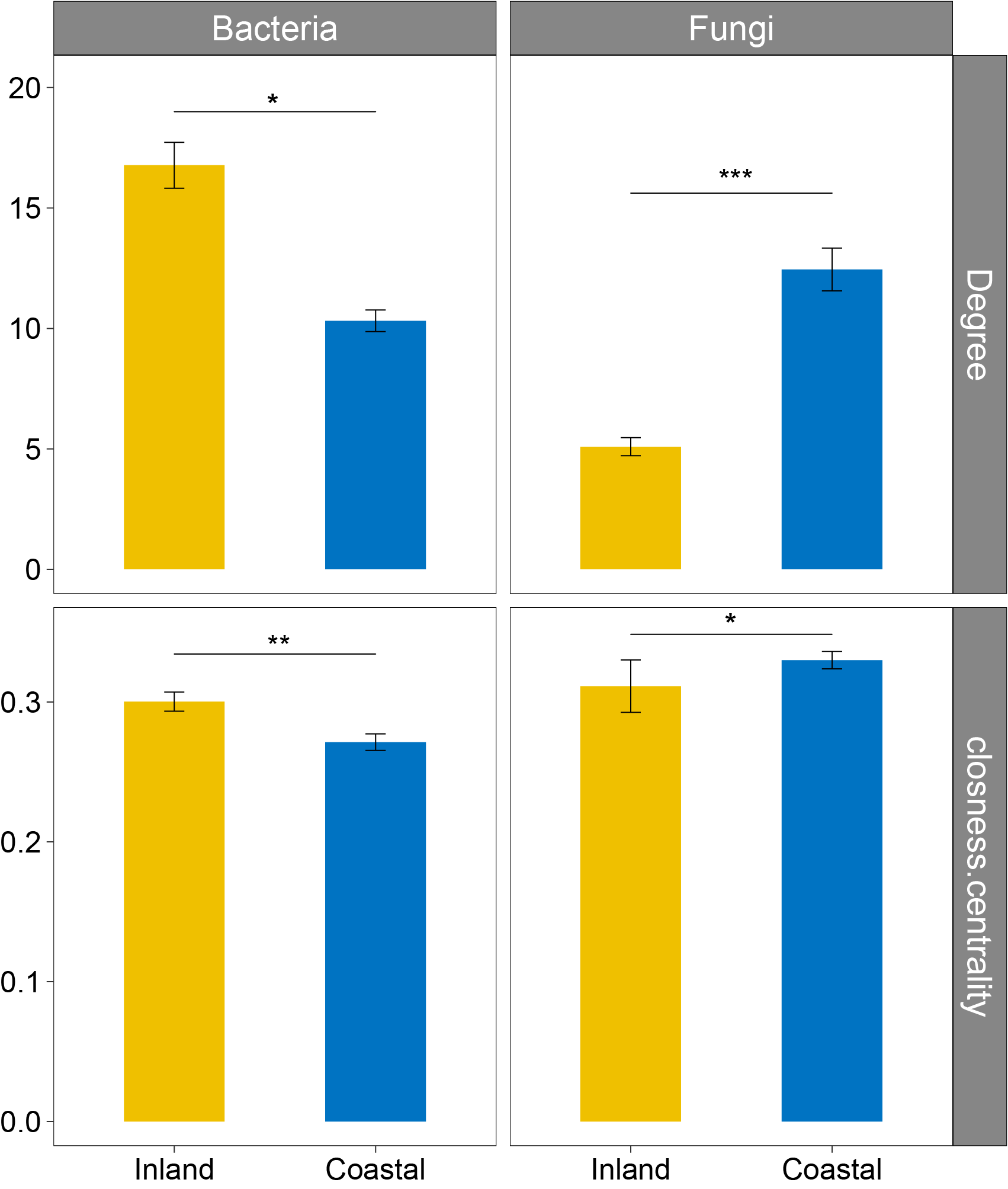


Fig. S4 Comparison of node-level topological features (degree and closeness centrality) of bacterial and fungal networks between inland and coastal regions. * *P* < 0.05. ** *P* < 0.01. *** *P* < 0.001.
